# A network of bat caves in Brazilian drylands support population connectivity in *Pteronotus* bats (Chiroptera: Mormoopidae)

**DOI:** 10.1186/s12862-025-02465-w

**Published:** 2025-11-24

**Authors:** Fernanda Ito, Veronika N. Laine, Enrico Bernard, Thomas M. Lilley

**Affiliations:** 1 Laboratório de Ciência Aplicada à Conservação da Biodiversidade, Departamento de Zoologia, Universidade Federal de Pernambuco, Recife, Pernambuco Brazil; 2 Programa de Pós-Graduação em Biologia Animal, Universidade Federal de Pernambuco, Recife, Pernambuco Brazil; 3 BatLab Finland, Finnish Museum of Natural History, University of Helsinki, Helsinki, Finland

**Keywords:** ddRADseq, Population structure, Genetic connectivity, Cave biota, Cave conservation, Landscape genetics, Tropical dry forests

## Abstract

*Pteronotus* is a Neotropical genus of mormoopid bats known to form large colonies in caves. Cave selection by *Pteronotus* is not random, and maternity roosts are characterized by the presence of a hot chamber where females give birth and raise their young. Such bat caves are unique from ecological and conservationist perspectives. Previous studies on bat caves in the drylands of Brazilian northeastern region – some holding up to 150,000 bats – have shown that *P. gymnonotus* presented no population structuring, with no correlation between geographical and genetic distance, and that bat caves up to 700 km apart form a very dynamic roost network used by that species. *P. personatus* is a smaller relative found in sympatry with *P. gymnonotus.* Both species frequently share the same roosts and are exposed to similar environmental conditions when foraging; therefore, they are good candidates for a comparison of their population genetic structuring. Using ddRADseq methodology, 55,836 SNPs from 72 *P. personatus* (21 females and 51 males) and 37,037 SNPs from 177 *P. gymnonotus* (93 females and 84 males) were analyzed. We observed that both species presented no strong signs of population genetic structure in the drylands of northeastern Brazil, and geographic distance is not the main factor shaping their genetic connectivity. Furthermore, our results suggest that the two *Pteronotus* species studied are characterized by very dynamic roost use, meaning that individuals frequently switch among caves within a landscape rather than relying on a single permanent roost. Thus, indicating that conservation initiatives should not rely solely on single-site protection but rather adopt a landscape-scale perspective.

**Supplementary Information:**

The online version contains supplementary material available at 10.1186/s12862-025-02465-w.

## Introduction

Globally, caves harbor an exceptional biodiversity and provide shelter for several endemic and threatened species (e.g [1–3]). Besides being the most used roost by bats, caves are also considered hotspots of invertebrate diversity and are critical to the survival of several species [2, 4]. Some of these habitats are known to support exceptionally large populations of bats: in the United States, the caves at Carlsbad Caverns National Park (New Mexico) and Bracken Cave (Texas) host populations estimated at over 5 million individuals of *Tadarida brasiliensis* [5]; In Malaysia, the Deer Cave at Gunung Mulu National Park is estimated to contain around 2 million *Chaerophon plicata* [5]. These caves are known as bat caves [6, 7] and some of them likely harbor the largest mammal congregations in the world (Mittermeier et al. 2003). Despite all this, caves are globally considered more vulnerable to anthropogenic impacts than other ecosystems and are often neglected in conservation planning [3, 8].

In the Neotropics some bat caves are formed by *Pteronotus* bats [9]. Commonly known as mustached and naked-backed bats, the genus *Pteronotus* comprises exclusively insectivorous, strictly cave-dwelling bats from the Mormoopidae family [10, 11]. Restricted to the Neotropics, eight species are currently recognized in the genus [10, 12], but the discovery of divergent lineages suggests that the diversity of this genus is underestimated [13, 14]. All species are gregarious, and many of them are broadly sympatric, commonly observed roosting together [10, 12, 15]. Cave selection by *Pteronotus* bats is not random, and maternity roosts are characterized by the presence of a hot chamber where females give birth and raise their young for 2–3 months [9, 16]. In Northeastern Brazil, *P. personatus* and *P. gymnonotus* form exceptionally dense colonies in bat caves, with up to 150,000 individuals in some of them [17–20]. Recent studies have shown that the large colonies in bat caves can alter the cave temperature with direct and indirect effects on other species [9], making *Pteronotus* a good candidate for key- and umbrella-species for cave conservation.

From a conservation perspective, quantifying connectivity among populations has become increasingly recognized as a priority for the conservation of species (e.g [21, 22]. , because small, isolated populations have higher risks of local extinction [23, 24], particularly in a scenario of intense anthropogenic impacts [22, 25]. The conservation of caves and their biotas summarizes such challenges: Cave organisms frequently present highly specialized characteristics not found in the outside habitats, they tend to have high endemism – sometimes restricted to a single cave – for most of them, mobility between different caves is extremely limited, and their habitats are inserted in highly modified human-altered matrices [26].

Despite the recognized importance of understanding population connectivity for cave [27]particularly in Brazil— remains limited. *Pteronotus* bats, with their large and dense colonies, are particularly vulnerable to environmental changes and human impacts, making them ideal candidates for genetic diversity studies. Only recently has the genetic diversity of these cave-dwelling bats been assessed in Brazil [27]. That study showed that *P. gymnonotus* presented no signs of population structuring, and that the geographical distance between the bat caves was not correlated with genetic distance between the populations, suggesting that a network of bat caves up to 700 km apart are used by that species [27]. Nonetheless, in the same region, *P. gymnonotus* is often reported to roost in association with *P. personatus*, also forming large colonies in bat caves (e.g [18–20, 28, 29]. In addition to being sympatric and roosting together, these two congeneric species also exhibit the similar patterns of fluctuation in their colony size [17, 19, 20], indicating the possibility of similarities in their reproductive patterns [27].

Although phylogenetically close, found in sympatry, and frequently sharing roosts under similar environmental conditions, *Pteronotus personatus* and *P. gymnonotus* differ, for example, in body size, evolutionary history, and habitat specificity [14, 15, 18, 30]. These similarities and differences make them ideal models for a comparative study of population genetic structuring. Understanding whether their genetic diversity and population structure reflect shared ecological pressures or are shaped by species-specific traits is essential, as this can provide insights into how ecological specialization influences genetic connectivity in cave-dependent bats. Moreover, because *Pteronotus* species form exceptionally dense colonies and play a key role in cave ecosystems, assessing their genetic diversity and connectivity is also critical from a conservation perspective. By analyzing the genetic structure of *P. personatus* across bat caves in Northeastern Brazil, and comparing these patterns with those previously described for *P. gymnonotus* [27], we aim to evaluate if ecological and evolutionary differences shape genetic patterns in two cave-dependent congeners, thereby contributing to both species-specific management and the broader conservation of bat cave ecosystems.

## Materials and methods

### Sample collection and library construction

**Fig. 1:**
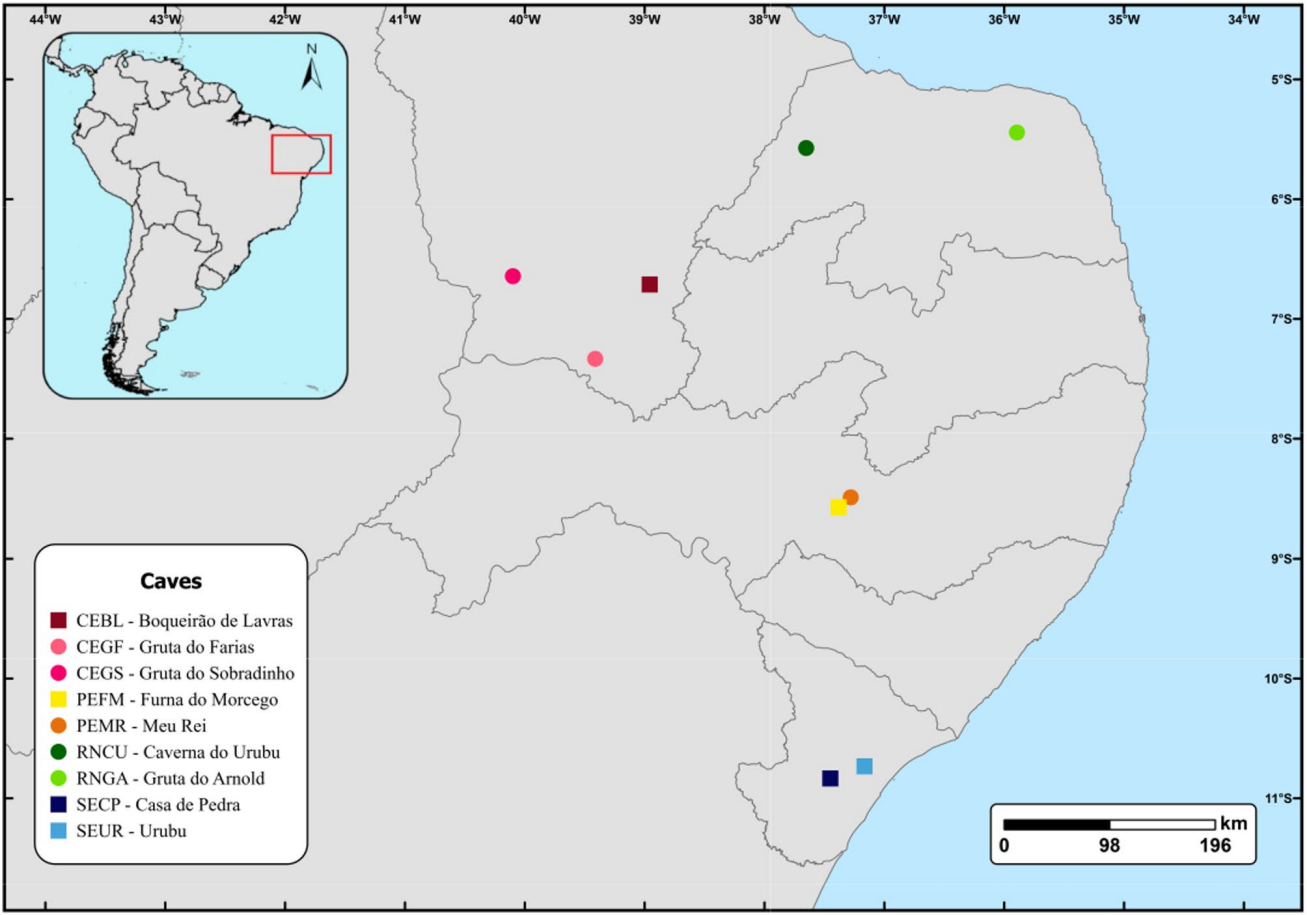
Map of bat caves in the Caatinga drylands, Northeast Brazil, where *Pteronotus* bats were sampled. Bat caves in the Caatinga drylands, Northeast Brazil, where *Pteronotus* bats were sampled. Bat caves where both *P. gymnonotus* and *P. personatus* were sampled are represented by colored squares, while the circles represent the caves where only *P. gymnonotus* was found

Nine bat caves located in the Brazilian states of Ceará, Rio Grande do Norte, Pernambuco, and Sergipe were sampled for *Pteronotus* bats from July 1 to July 28, 2019 (Fig. 1; Table 1). Colony sizes were estimated using a non-invasive thermal detection system as described by Otálora-Ardila et al. [17] and Barros and Bernard [9], with two counts conducted per cave. Bats were captured using a hand net inside the caves, and individuals were euthanized in accordance with the guidelines set by the American Society of Mammalogists [31]. Liver tissue samples were collected from approximately 20 adult individuals per species in each cave. Tissue samples were stored in 1.5 mL tubes filled with 95% ethanol on ice during collection, then transported and stored at -80 °C until further analysis. All voucher specimens were deposited in the Mammal Collection of the Federal University of Pernambuco (UFPE). This research was conducted under the SISBIO/ICMBio permit 68992-1, registered in SisGen under protocol A974BB7, and approved by the Commission on Ethics and Animal Use of the Federal University of Pernambuco (CEUA-UFPE 114/2019).

**Table 1:** Bat caves from the Brazilian Northeast where *Pteronotus* bats were sampled

Cave	Locality	Coordinates	Colony Size	*P*.gymnonotus		*P*.personatus	
				*N*	F/M	*N*	F/M
**RNGA**							
Gruta do Arnold	João Câmara/RN	05°26’36.2"S 35°53’37.1"W	5,365	21	11/10	-	-
**RNCU**							
Caverna do Urubu	Felipe Guerra/RN	05°34’22.8’’S 37°39’08.8"W	22,743	23	13/10	**-**	**-**
**CEGS**							
Gruta do Sobradinho	Aiuaba/CE	6°38’35.6"S 40°5’57.1"W	57,200	15	10/5	-	-
**CEBL**							
Boqueirão de Lavras	Lavras da Mangabeira/CE	06°42’45.05"S 38°57’28.1"W	86,089	22	17/5	23	5/18
**CEGF**							
Gruta do Farias	Arajara Park/CE	07°19’59.0"S 39°24’45.9"W	11,540	24	13/11	-	-
**PEMR**							
Meu Rei	PARNA Catimbau/PE	08°29’14.1’’S 37°16’48.8’’W	13,828	11	8/3	-	-
**PEFM**							
Furna do Morcego	PARNA Catimbau/PE	08°34’14.1’’S 37°22’55.4’’W	37,789	24	16/8	11	6/5
**SEUR**							
Urubu	Divina Pastora/SE	10°43’58.1"S 37°09’56.0"W	62,149	20	3/17	24	7/17
**SECP**							
Casa de Pedra	Campo do Brito/SE	10°50’03.0"S 37°27’03.6"W	98,986	17	2/15	14	3/11

The name of all caves follows the ones provided by ICMBio. Colony size represents an estimation of the number of bats present in the cave in the sampling night. The number of individuals collected (N) and the number of females (F) and males (M) individuals sampled are shown for each *Pteronotus* species. Data for *P. gymnonotus* as in Ito et al. [27]

Genomic DNA was extracted from liver tissue using the DNeasy Blood & Tissue Kit (QIAGEN, Inc.), following the manufacturer’s protocol. The DNA concentration was measured using a Thermo Scientific NanoDrop spectrophotometer, and all samples were diluted to a final concentration of 20 ng/µL, following the protocols of Lilley et al. [32] and Ito et al. [27]. DNA libraries were prepared using a double-digest RAD-seq (ddRAD) method, adapted from Lemopoulos et al. [33] and Elshire et al. [34] to accommodate low-concentration samples. Sequencing was performed by Bioname Oy on an Illumina Novaseq 6000 platform in a single lane [33, 34]. For *P. personatus*, paired-end sequencing was performed, generating 100 bp paired-end reads. The raw sequence data are available in the NCBI SRA archive under BioProject PRJNA956837. To ensure comparability between species, only the R1 reads from the paired-end sequencing of *P. personatus* were used in downstream analyses. In contrast, for *P. gymnonotus*, we reanalyzed raw sequence data originally generated by Ito et al. [27], available under BioProject PRJNA824143. In that study, single-end sequencing was performed, thus all single-end reads were processed using the same bioinformatic pipeline as for *P. personatus*, ensuring consistency in data handling.

### Sequence processing and SNP calling

Fastp 0.21.0 [35] was employed for demultiplexing, adapter trimming, and quality filtering of reads for both species. For *P. personatus*, the R1 reads from the paired-end sequencing were processed as single-end reads, while for *P. gymnonotus*, the single-end reads generated by Ito et al. [27] were processed directly. Quality trimming was performed using a sliding window approach with a Q20 threshold. Low-quality regions and uncalled bases were trimmed, applying a minimum read length filter of 30 bp for both species.

The trimmed reads were then processed through the de novo pipeline in Stacks 1.48 for SNP calling [36], using the complete dataset with all reads from both species. The pipeline parameters were configured to permit a maximum of three mismatches, ensuring rigorous filtering of potential SNPs. The populations module was employed to analyze genetic variation across 13 populations representing the species and the bat caves from which they were collected—four populations for *P. personatus* and nine for *P. gymnonotus* (Table 1). Subsequently, SNP filtering was conducted using vcftools 4.1 [37], with the following parameters: maf = 0.05, max-missing = 0.7, min-meanDP = 10; max-meanDP = 100.

For comparative purposes, we also ran the de novo pipeline for each species separately with the same parameters, after testing several combinations of parameter settings [38] and assigning the samples to the caves where they were collected. The complete scripts used for data processing and analysis are available on Zenodo.

### Data analysis

The final dataset, which included all samples from both species, was then used on the *populations* pipeline from Stacks 1.48 [36] to estimate the genetic diversity indices for each subpopulation, which included number of private alleles (N_A_), nucleotide diversity (π), inbreeding coefficient (F_IS_), and the expected (H_E_) and observed (H_O_) heterozygosity. Additionally, these estimations were conducted separately for each species to assess species-specific genetic diversity. Principal Component Analyses (PCA) were performed using Plink 1.09 [39] for the combined dataset, which included all individuals from both *Pteronotus* species, as well as for each species separately.

The inference of individual ancestry coefficients was conducted using ADMIXTURE 1.3.0, which is based on likelihood models with quasi-Newton convergence acceleration method [40]. The ADMIXTURE for the dataset that included all specimens from both *P. personatus* and *P. gymnonotus* species, possible K values were evaluated ranging from one up to nine and 10 runs were performed for each number of K. For *P. personatus* dataset, ADMIXTURE analysis considered each of the four caves as a population, and 10 runs were performed for values of the number of clusters set to K = 1–4. While for the *P. gymnonotus* dataset, the analysis considered each of the nine caves as a population, and 10 runs were performed for values of the number of clusters set to K = 1–9. The cross-validation error (CV error) was used to select the best K value [40].

The pairwise Weir and Cockerham weighted F_ST_ estimates [41] were calculated for each species separately with vcftools v.0.1.17 [37], using each cave as a population. F_ST_ is a standardized variance that represents the portion of the total genetic variance that is due to among-subpopulation differences [42]. F_ST_ values of 0 to 0.05 were considered to be of low differentiation, and 0.05 to 0.15 as moderate differentiation, whereas F_ST_ values >0.15 were considered distinctly differentiated [42]. The latitude and longitude coordinates of the sampling locations were used to calculate pairwise geographic distances between caves in kilometers using the Haversine method assuming a spherical earth, implemented in the function *distm* in the R package geosphere 1.5.14 [43]. Isolation-by-distance was tested for each species with a Mantel test with complete permutations, using the pairwise F_ST_ as a measure for the genetic distances and the between-cave distances as geographic coordinates for populations, with 10,000 permutations and considering alfa = 0.05.

## Results

### Colony sizes and species occurrence

*Pteronotus personatus* was found in four caves (Boqueirão de Lavras, Furna do Morcego, Urubu, and Casa de Pedra), whereas *P. gymnonotus* occurred in all nine caves sampled (Table 1). Colony size estimates confirmed large bat populations in all caves, ranging from 5,365 bats at Gruta do Arnold to 98,986 bats at Casa de Pedra (Table 1). When considering only the caves where both species occurred, colony sizes ranged from 37,789 bats at Furna do Morcego to 98,986 bats at Casa de Pedra. The three largest colonies were found in caves harboring both *Pteronotus* species—Casa de Pedra, Boqueirão de Lavras, and Urubu (Table 1). Colonies in these caves are formed mainly by *P. gymnonotus* and *P. personatus*, and based on echolocation records [20], we considered an approximate 50/50 distribution between the two species when sharing roosts. In contrast, the three smallest colonies, at Gruta do Arnold, Gruta do Farias, and Meu Rei, were formed exclusively by *P. gymnonotus* (Table 1).

### Genetic diversity and population structure

To allow direct comparison between species, the *P. personatus* dataset represents newly generated data from this study, while the *P. gymnonotus* dataset consists of raw reads originally produced by Ito et al. [27] that were reanalyzed here using the same bioinformatic pipeline. This reanalysis ensures methodological consistency between the two species and provides new results for *P. gymnonotus* that are directly comparable to those of *P. personatus*.

The combined dataset comprised 249 samples from both *Pteronotus* species: 72 individuals from *P. personatus* (21 females and 51 males) and 177 individuals from *P. gymnonotus* (93 females and 84 males; Table 1; [27]). After quality filtering, SNP calling, and filtering, the combined dataset comprised 16,599 SNPs across 249 individuals (Table 1). In the separate SNP call datasets, the *P. personatus* dataset contained 55,836 biallelic SNPs from 72 individuals from four bat caves, while the *P. gymnonotus* dataset consisted of 37,037 SNPs from 177 individuals from nine bat caves.

Principal Component Analysis (PCA) results based on the complete dataset with all samples of *Pteronotus* showed no overlap between *P. personatus* and *P. gymnonotus* (Figure S1), confirming that the samples belong to two different species and were correctly identified on the field. The first two principal components of the analysis explained 95.69% of the total variation and separated the 249 individuals into two clusters, one for each species (Figure S1). Similarly, the ADMIXTURE results indicated two distinct clusters, formed by individuals of each species (Figure S1).

**Fig. 2:**
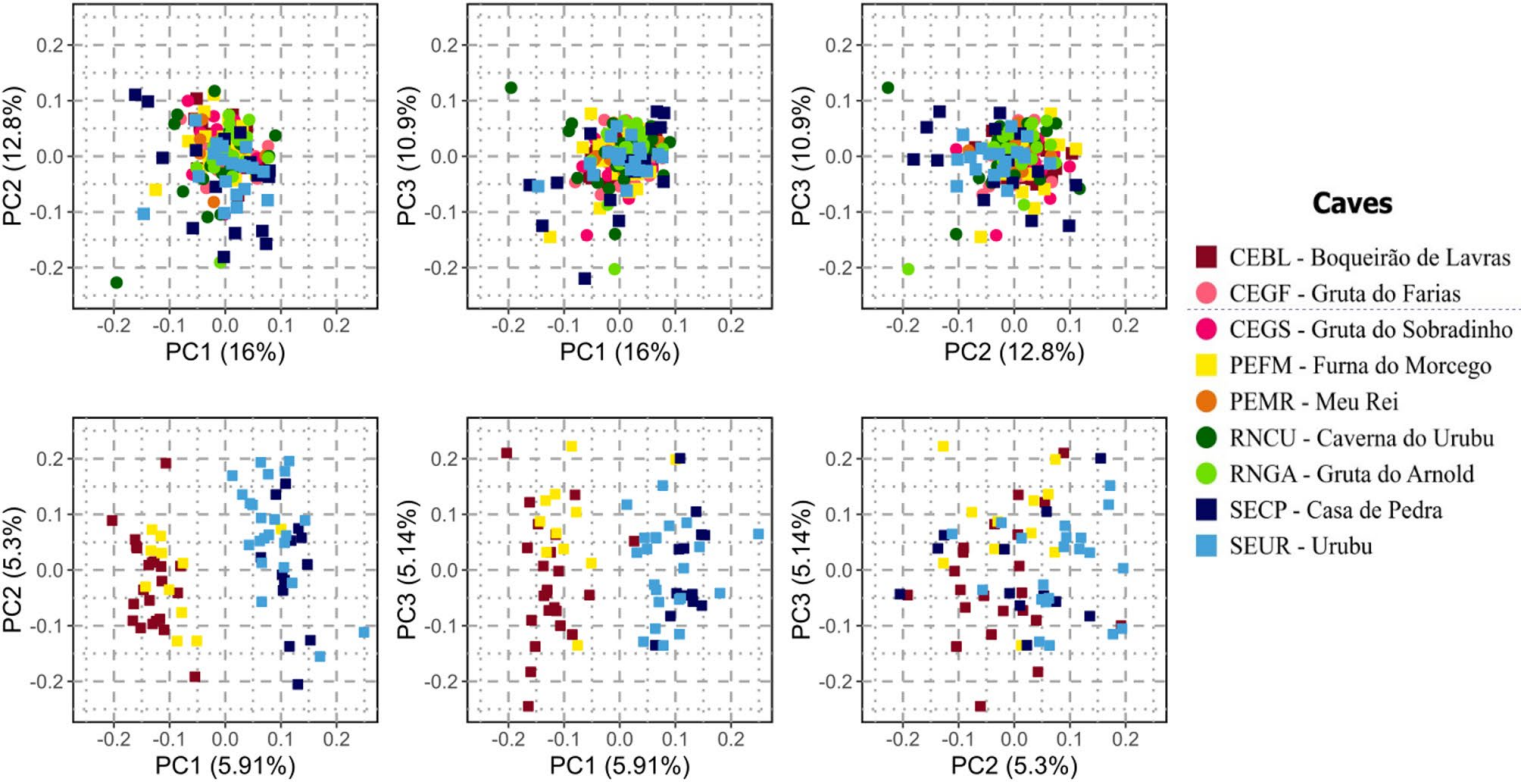
Principal Component Analysis (PCA) of *Pteronotus* bats. Population genetic structure of *Pteronotus* bats from the bat caves in Northeastern Brazil represented by a Principal Component Analysis (PCA). On the top row are the PCA results for 177 individuals of *Pteronotus gymnonotus* from nine bat caves. Bottom row results are for 72 individuals of *Pteronotus personatus* sampled on four bat caves

Based on the separate datasets, genetic diversity metrics for *P. personatus* indicated nucleotide diversity (π) between 0.256 and 0.258 and observed heterozygosity (H_O_) between 0.236 and 0.239 across the four caves where the species can be found, while inbreeding coefficient values (F_IS_) were higher (0.059–0.081) than in *P. gymnonotus*, suggesting more pronounced inbreeding in this species (Table 2). PCA of *P. personatus* samples (72 individuals) revealed that PC1 and PC2 explained 11.21% of the genetic variation, while PC1 and PC3 explained 11.05% and PCs 2 and 3 accounted for 10.44% (Fig. 2). The results show no strong pattern of genetic structure, although the first two components roughly separate individuals into two clusters (Fig. 2). ADMIXTURE analyses also supported the absence of structure, with K = 1 identified as the best K value (Figure S2). Pairwise F_ST_ values were low (< 0.05), supporting a lack of strong differentiation (Table 3). The lowest F_ST_ value was between Casa de Pedra (SECP) and Urubu (SEUR; F_ST_ = 0.0005), while the highest was between Casa de Pedra and Boqueirão de Lavras (SECP × CEBL; F_ST_ = 0.0049), followed closely by Casa de Pedra and Furna do Morcego (SECP × PEFM; F_ST_ = 0.0048; Table 3). F_ST_ values did not correspond to geographical distances: for example, Furna do Morcego (PEFM) and Boqueirão de Lavras (CEBL) had a much lower F_ST_ than Furna do Morcego and the southern caves, despite being geographically more distant. The Mantel test revealed a positive but non-significant correlation between genetic and geographic distances (*r* = 0.7321, *p* = 0.1119), indicating that geographic distance alone does not explain the genetic patterns observed in the studied populations.

**Table 2:** Genetic diversity of *Pteronotus* Bats from bat caves in the Brazilian Northeast

	*P*. gymnonotus				*P*. personatus			
**Cave**	**π**	**F**_**IS**_	**Heterozygosity**		**π**	**F**_**IS**_	**Heterozygosity**	
			**H**_**O**_	**H**_**E**_			**H**_**O**_	**H**_**E**_
RNGA - Gruta do Arnold	0.256	0.047	0.253	0.267	-	-	-	-
RNCU - Caverna do Urubu	0.254	0.054	0.249	0.265	-	-	-	-
CEGS - Gruta do Sobradinho	0.255	0.001	0.266	0.265	-	-	-	-
CEBL – Boqueirão de Lavras	0.256	0.021	0.260	0.265	0.257	0.081	0.236	0.260
CEGF - Gruta do Farias	0.255	-0.021	0.274	0.264	-	-	-	-
PEMR - Meu Rei	0.253	-0.001	0.262	0.262	-	-	-	-
PEFM - Furna do Morcego	0.254	0.026	0.259	0.266	0.258	0.069	0.237	0.266
SEUR - Urubu	0.254	0.065	0.246	0.266	0.256	0.074	0.239	0.261
SECP - Casa de Pedra	0.220	0.068	0.204	0.226	0.258	0.059	0.237	0.256

Genetic diversity of *Pteronotus* bats from bat caves in the Brazilian Northeast, including nucleotide diversity (π), inbreeding coefficient (F_IS_), and the expected (H_E_) and observed (H_O_) heterozygosity. Data for *P. personatus* is based on 55,836 SNPs from 72 individuals from four caves, and data for *P. gymnonotus* is based on 37,037 SNPs from 177 individuals from all nine caves

**Table 3:** Pairwise F_ST_ estimate and geographic distance in Kilometers for *Pteronotus personatus*

	CEBL	PEFM	SEUR	SECP
CEBL	-	0.0009	0.0041	0.0049
PEFM	270	-	0.0026	0.0048
SEUR	489	241	-	0.0005
SECP	487	252	33	-

Pairwise Weir and Cockerham weighted F_ST_ estimate (above diagonal) and geographic distance in kilometers (below diagonal) for *Pteronotus personatus* bats sampled in four different bat caves in Northeastern Brazil. Cave name acronyms as in Table 1

For *P. gymnonotus*, genetic diversity was moderate and relatively homogeneous across caves, with nucleotide diversity (π) values ranging from 0.220 in SECP to 0.256 in multiple caves. Observed heterozygosity (H_O_) was generally similar to expected heterozygosity (H_E_), and inbreeding coefficients (F_IS_) were low and mostly positive, indicating slight but consistent inbreeding, except at CEGF and PEMR where values were slightly negative (Table 2). PCA showed that PC1 and PC2 explained 28.8% of the genetic variation, while PCs 1 and 3 and 2 and 3 explained 26.9% and 23.7% respectively (Fig. 2). No genetic structure was observed across the nine caves, suggesting a single panmictic population. ADMIXTURE analyses similarly supported K = 1 as the best fit, with all individuals belonging to the same ancestral population (Figure S2). Pairwise F_ST_ values were even lower than those of *P. personatus*, including negative values (Table 4). The lowest value was between Caverna do Urubu (RNCU) and Casa de Pedra (SECP; F_ST_ = − 0.0075), while the highest was between Gruta do Arnold (RNGA) and Gruta do Farias (CEGF; F_ST_ = 0.0014). Consistent with the lack of genetic structure observed in the species, the Mantel test revealed a negative, non-significant correlation (*r* = − 0.1445, *p* = 0.7982), which suggests no significant correlations between genetic and geographic distances.

**Table 4:** Pairwise F_ST_ estimate and geographic distance in Kilometers for *Pteronotus gymnonotus*

	RNGA	RNCU	CEGS	CEBL	CEGF	PEMR	PEFM	SEUR	SECP
RNGA	-	0.0002	0.0005	-0.0005	0.0014	-0.0001	0.0010	0.0007	0.0006
RNCU	195	-	0.0005	0.0008	0.0004	0.0000	0.0006	0.0004	-0.0075
CEGS	484	295	-	-0.0001	0.0007	0.0011	-0.0046	0.0004	0.0003
CEBL	367	192	126	-	0.0006	-0.0033	0.0001	0.0006	0.0008
CEGF	442	276	108	85	-	0.0012	-0.0006	-0.0037	0.0009
PEMR	371	327	372	270	268	-	0.0001	-0.0002	0.0001
PEFM	385	335	368	270	262	15	-	0.0002	-0.0006
SEUR	605	576	550	489	452	250	241	-	-0.0002
SECP	624	586	550	487	445	261	252	33	-

Pairwise Weir and Cockerham weighted F_ST_ estimate (above diagonal) and geographic distance in kilometers (below diagonal) for *Pteronotus gymnonotus* bats sampled in nine different bat caves in Northeastern Brazil. Cave name acronyms as in Table 1. Four of these caves also host *P. personatus* populations (see Table 1)

Overall, both *Pteronotus* species showed relatively homogeneous levels of genetic diversity across sampled caves, with *P. personatus* presenting slightly higher inbreeding coefficients than *P. gymnonotus*. Pairwise *F*_ST_ values were consistently low, and neither PCA, ADMIXTURE, nor Mantel tests indicated significant population subdivision. Furthermore, genetic diversity for the species does not directly correlate to the geographical distances among caves. Thus, these results suggest that *Pteronotus* populations in the Caatinga bat caves form largely panmictic units, with no evidence of strong genetic structuring.

## Discussion

In this study, we explored, for the first time in Brazil, the population genetic structure of two sympatric bat species, *Pteronotus personatus* and *Pteronotus gymnonotus*, both cave-dependent taxa. Our findings indicate that in the Caatinga drylands, *P. personatus* was restricted to four bat caves, whereas *P. gymnonotus* was more widely distributed, occurring in all surveyed caves. Despite these differences in occurrence, both species exhibited relatively homogeneous levels of genetic diversity and no clear evidence of population structuring. For *P. personatus*, we observed only weak signs of genetic differentiation among caves, which were not clearly associated with geographical distance across analyses, suggesting that while some differentiation may be emerging, gene flow still occurs among caves, maintaining overall connectivity. In contrast, *P. gymnonotus* cave subpopulations showed high levels of connectivity even across large distances. Importantly, no hybridization was detected between the two species, underscoring their distinct genetic identities.

Bats of the genus *Pteronotus* are cave specialists, exhibiting a strict relationship with their roosts [9, 30]. They typically select caves with high climatic stability, low air circulation, and high temperature and relative humidity [9]. Hot chambers within these caves, where temperatures can reach up to 40 °C and relative humidity is ≥ 90%, are crucial for the development of young bats and the maintenance of a relatively high and constant body temperature [9, 16, 30]. Such specific habitat requirements reinforce the importance of those bat caves, which may be considered exceptional ecological sites [27, 44] and unique environments due to a combination of biotic and abiotic interactions [6, 7, 45]. Although caves with exceptional bat populations are present in countries across South, Central, and North America (e.g [6, 7], those holding *Pteronotus* populations are not abundant. In Brazil’s Caatinga drylands, only ten bat caves are known (E. Bernard, personal communication), and the nine caves surveyed here are the only ones with *Pteronotus* bats. Thus, our results not only encompass all Pteronotus colonies regionally known to date but also reinforce the strict roost dependency of these species, and highlighting the urgent need to protect these habitats and their ecological functions.

In Brazil, the ranges of *P. personatus* and *P. gymnonotus* broadly overlap [15], and in the Caatinga drylands, they frequently share roosts (e.g [9, 18]). However, *P. personatus* was found in only four of the nine surveyed caves, highlighting its more restricted distribution compared to *P. gymnonotus*, which occurred in all caves. This restricted distribution of *P. personatus* is consistent with bioacoustic and ecological data (e.g [9, 20, 46]) and may reflect narrower habitat preferences or ecological constraints not faced by *P. gymnonotus*. Ecological theory predicts that species with wider distributions and higher local abundance function as generalists, exhibiting higher gene flow and lower genetic structuring [47]. In contrast, specialist species are typically rarer and more prone to population differentiation [47, 48]. Within this framework, our results suggest that *P. gymnonotus* behaves as a generalist, with high abundance and genetic connectivity across caves, while *P. personatus* shows traits of a more specialized species. Nonetheless, contrary to expectations for specialists, *P. personatus* in the Caatinga only exhibited weak signs of genetic structuration. This suggests that other ecological or behavioral traits may help maintain connectivity across its more limited distribution, and stronger structuring can be observed across its broader range or under scenarios of habitat fragmentation.

Additionally, our results suggest differences in habitat use and ecological tolerance, highlighting the importance of species-specific ecological dynamics in shaping population genetic structure. The restricted distribution of *P. personatus*, recorded in only four of the nine surveyed caves, indicates potential ecological limitations. These may be associated with habitat specificity, interspecific competition, or other ecological constraints that limit its occurrence to fewer roosting sites. *P. personatus* appears to rely heavily on the hot chambers of bat caves for reproduction [16], which may reduce its dispersal capacity and increase its susceptibility to habitat fragmentation. Such ecological specialization can influence movement patterns and promote genetic isolation among subpopulations, a pattern also observed in other taxa such as plants (e.g [49]), invertebrates (e.g [50]), birds (e.g [51]), lizards (e.g [52]), and bats (e.g [53]). Although some studies indicate that gene flow may still occur at fine spatial scales among ecologically similar sites (e.g [51, 54]), three lines of evidence suggest that *P. personatus* may still exhibit strong population genetic structure across its full distribution range: (1) its lower local abundance, with presence confirmed in only a subset of caves; (2) its apparent dependence on specific environmental conditions; and (3) the patterns of genetic diversity observed in this study. These factors, considered together, highlight the importance of broader-scale studies to fully understand the population dynamics and conservation needs of this species.

In contrast, the widespread presence of *P. gymnonotus* across all surveyed caves suggests that this species has a broad ecological tolerance. In fact, this species is distributed across a wide range of habitats in the Neotropics, from southeastern Mexico through Central and South America, including northeastern Brazil [18]. However, it is important to note that this abundance pattern applies to the species’ southern range. In the northernmost distribution, populations are much smaller, with colonies of fewer than 30 individuals [55]. Additionally, as an aerial insectivore, *P. gymnonotus* forages in open areas and gallery forests, further demonstrating its ability to exploit diverse habitats [18]. This ecological flexibility suggests a high level of adaptability and likely contributes to its high genetic connectivity and resilience to environmental changes. Together with the restricted distribution of *P. personatus* in the Caatinga, these contrasting patterns highlight the complexity of ecological dynamics within the genus and reinforce the need for a landscape-scale approach to conservation.

Furthermore, the absence of hybridization between *P. gymnonotus* and *P. personatus*, despite their frequent co-roosting, underscores their distinct evolutionary identities and potential ecological partitioning. Hybridization events in bats have been documented in other contexts, such as between *P. gymnonotus* and *P. fulvus* in Mexico, where introgression occurred between two closely related and sympatric species [56], and in *Myotis* bats, particularly at swarming sites where large mixed colonies form [57, 58]. These cases demonstrate that while hybridization is possible within the genus or among closely related bat taxa, it does not occur between *P. gymnonotus* and *P. personatus*, further emphasizing the complexity of their coexistence and distinct ecological trajectories.

Globally, multiple studies have already reported the impacts of small isolated populations and low levels of genetic diversity on species´ persistence [22, 59, 60]. However, other studies have shown that genetic diversity alone is not enough to fully assess the conservation status of a species [60] and the maintenance of gene flow among populations is essential to safeguard the species´ long-term survival [22, 61]. Therefore, identifying and understanding the drivers of gene flow is imperative for species conservation. Here, the analysis of the population genetic structure of two *Pteronotus* species suggests that although geographic distance may influence gene flow, the patterns we observed do not constitute a clear case of Isolation by Distance. Pairwise *F*_ST_ values and Mantel test results indicate ongoing connectivity among caves, especially for *P. gymnonotus* and, to a slightly lesser extent, for *P. personatus*. Since geography is only one of the key components that can influence population connectivity [62, 63], other factors – or a combination of them – could be driving the population genetic structure in the species (e.g [53, 64]), which appears to be the case for the two *Pteronotus* species we studied.

Furthermore, population genomic studies of bats using high-resolution markers such as SNPs or UCEs are extremely rare, making comparative analyses crucial for understanding dispersal ecology and the drivers of population structure. Alongside our genomic analyses of *Pteronotus gymnonotus*, a key example is the study by Lilley et al. [32] on the Chilean *Myotis*, *Myotis chiloensis*, which also employed ddRAD-seq to analyze genome-wide SNPs. In *M. chiloensis*, populations exhibited strong genetic structure and pronounced isolation-by-distance, with higher genetic diversity observed in southern populations (H_O_ up to 0.3248). In contrast, *Pteronotus* bats in the Caatinga displayed low genetic differentiation (F_ST_ generally < 0.05), and no significant correlation between genetic and geographic distance. Heterozygosity values further highlight these contrasts: H_O_ ranged from 0.236 to 0.239 and H_E_ 0.256–0.266 for *P. personatus*, and H_O_ 0.204–0.274 and H_E_ 0.226–0.267 for *P. gymnonotus*, whereas *M. chiloensis* populations reached H_O_ 0.255–0.3248 and H_E_ 0.279–0.3335. Similarly, inbreeding coefficients were consistently higher in *P. personatus* (F_IS_ = 0.059–0.081) compared with the low or slightly negative values observed in *P. gymnonotus*, while *M. chiloensis* populations exhibited F_IS_ ranging from 0.021 to 0.058. Notably, the geographic patterns of diversity differ between these genera: in *M. chiloensis*, genetic diversity increases from north to south, with strong population differentiation (F_ST_ up to 0.113) and clear isolation-by-distance, whereas both *Pteronotus* species show relatively homogeneous diversity across caves, low F_ST_ values, and no significant correlation between genetic and geographic distance. These contrasts suggest that *Pteronotus* populations in the Caatinga form largely panmictic units, maintaining high connectivity via dynamic roost use and presumed long-distance mating movements across the cave network, in stark contrast to the highly structured, spatially isolated populations of *M. chiloensis*.

For many species, gene flow is not a direct result of migration to a new population. Instead, it can occur through mating events, when individuals temporarily disperse to mate and then return to their original populations [65–68]. In these cases, bats congregate to mate at swarming sites, promoting genetic mixing among populations (e.g [69–71]). These temporary movements can be driven by either female and/or male dispersal and may or may not have a clear seasonal pattern [66, 70, 72]. In fact, we [27] have already suggested that movements related to reproduction are the main factor shaping population genetic structure in *P. gymnonotus*. The lack of population structure and the high level of genetic diversity for *P. personatus* also suggest that reproductive strategies play an important role. This would explain the level of genetic connectivity between populations of geographically distant caves. Previous studies have reported nursery colonies and movement of adult male individuals among bat caves for *P. gymnonotus* [17, 19, 20, 73], which may explain the level of connectivity among all nine subpopulations studied.

Taken together, these results reinforce that, for *P. personatus*, the weak signals of population structuring we observed are not indicative of fully differentiated clusters but rather suggest high connectivity among the caves studied. However, given that we only sampled a portion of the species’ distribution and the ecological drivers of dispersal and roost selection remain largely unknown, broader landscape-scale studies are needed to understand potential future structuring. In this context, conserving the network of bat caves is critical to maintaining genetic diversity and connectivity, ensuring long-term population persistence for both *Pteronotus* species.

### Conservation implications

Our results emphasizes that *Pteronotus* species in northeastern Brazil have a very dynamic roost use, based on a network of caves, pointing out that conservation initiatives for the genus must not be based solely on a single site protection approach but, rather, on a landscape perspective. Cave management and conservation plans should consider the genetic information produced, and bat caves in Brazil must be managed as a network of roosts harboring very mobile individuals and hotspots for gene flow (e.g [7]). *Pteronotus personatus* was found in only four caves (Boqueirão de Lavras, Furna do Morcego, Urubu and Casa de Pedra), pointing out that such caves are extremely important as reproductive sites. The caves sheltering only *P. gymnonotus* are similarly important for bat conservation and must be protected, since they form a network of shelters, and should be studied closely to identify the reasons for *P. personatus* absence.

Our data highlight the need for further in-depth ecological studies on cave-dwelling bats in Brazil. In the case of *P. personatus*, for example, a taxonomic review is imperative considering that studies including molecular and morphological data have pointed out the possibility of a species complex [14, 74, 75], but with a pending description of the possible species. Similarly, updated data on the species distribution range and reproductive patterns are important and would help on the interpretation of the current population genetics results.

Finally, the evidence of long-distance mating movements presented here, especially for *P. gymnonotus*, together with the signs of structuration for *P. personatus*, highlights the importance of adopting a landscape genetics perspective for bat and cave conservation in the Caatinga. Even low levels of genetic differentiation underscore the need to preserve the entire network of roosts to maintain connectivity and safeguard genetic diversity. Current evidence has shown that the Caatinga is, in fact, a very dynamic and heterogeneous system, shaped by multiple ecological processes at different spatial and temporal scales along its area [76], and anthropogenic disturbance is unevenly distributed across the landscape [77], negatively impacting the bat activity in the region [78]. Thus, the landscape around the bat caves studied is under different degrees of anthropic pressure and, due to the complex use the different bat species make of them, both could be negatively impacted by habitat loss and degradation.

Considering the results presented here, both species of *Pteronotus* have a strict relationship with the bat caves. Thus, maintaining the genetic connectivity among the caves is essential for both species’ survival and the cave ecosystem. *Pteronotus* bats have been identified as an umbrella-taxa for both bats and caves in Brazil (e.g [9]). Their presence can influence bat diversity, including threatened species [9, 79]. The guano they produce is essential for the maintenance of several endemic cave species [80] and some subterranean ecosystems. In fact, their role as bioengineers has been recently addressed in Amazonian iron ore caves [45]. Moreover, bats in bat caves provide many other ecosystem services, such as the control of arthropod populations [20]. Therefore, setting the network of bat caves as priority for conservation would benefit both bats, the ecosystem services they provide, and the general speleological heritage in Brazil.

## Supplementary Information

Below is the link to the electronic supplementary material.

Supplementary Material 1: Figure S1. Sampling sites and population genetic results for the complete dataset of Pteronotus personatus and P. gymnonotus. (a) Map of bat caves sampled in Northeastern Brazil. Caves with both species are shown as squares, while caves with only *P. gymnonotus* are shown as circles. (b) Principal Component Analysis (PCA) of 249 individuals. Each point represents a bat, colored by sampling cave; squares denote *P. personatus* and circles denote *P. gymnonotus*. (c) ADMIXTURE analysis of the same individuals. Each vertical bar represents one individual, grouped by species (PP = *P. personatus*, PG = *P. gymnonotus*) and cave, with colors indicating ancestral population assignment. The lowest CV error was recovered for K = 3.

Supplementary Material 2: Figure S2. ADMIXTURE results for P. gymnonotus and P. personatus. ADMIXTURE results for K=2 and K=3 for *Pteronotus gymnonotus* (top) and *Pteronotus personatus* (bottom) from bat caves in Northeast Brazil. Individuals are represented by horizontal bars, grouped according to the caves where they were sampled.

## Data Availability

The datasets presented in this study can be found in the NCBI SRA repository, under SRA BioProjects PRJNA956837 and PRJNA824143, and the complete scripts for the analyses performed are available on Zenodo.
