# Supplementary figures and images for: A network of bat caves in Brazilian drylands support population connectivity in Pteronotus bats (Chiroptera: Mormoopidae)
Source: BMC Ecol Evol. 2025 Nov 24;25:128. doi: 10.1186/s12862-025-02465-w (PMC12642343; doi:10.1186/s12862-025-02465-w)

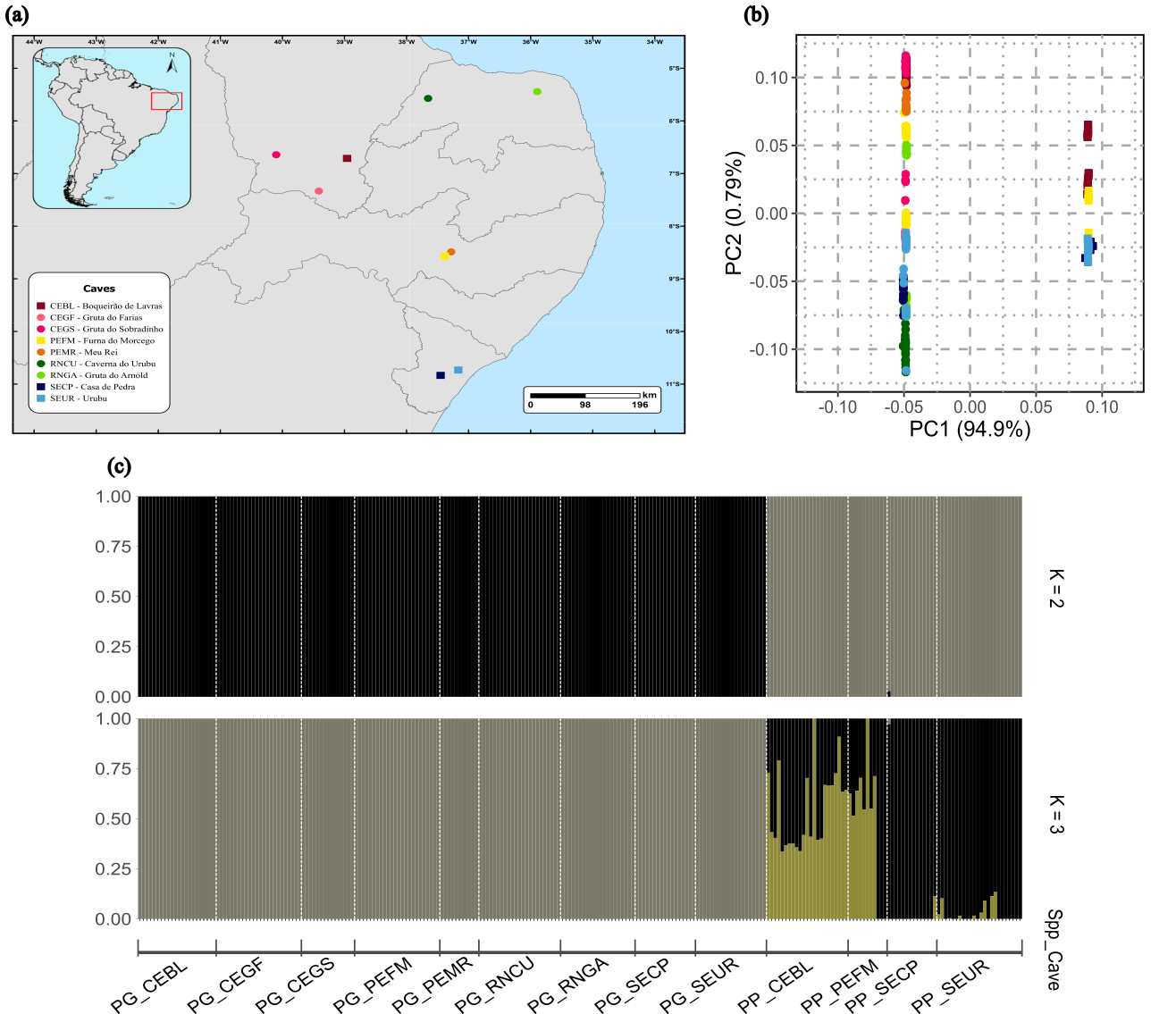

Supplement: Supplementary file 1 — Supplementary Material 1: Figure S1. Sampling sites and population genetic results for the complete dataset of Pteronotus personatus and P. gymnonotus. (a) Map of bat caves sampled in Northeastern Brazil. Caves with both species are shown as squares, while caves with only P. gymnonotus are shown as circles. (b) Principal Component Analysis (PCA) of 249 individuals. Each point represents a bat, colored by sampling cave; squares denote P. personatus and circles denote P. gymnonotus. (c) ADMIXTURE analysis of the same individuals. Each vertical bar represents one individual, grouped by species (PP = P. personatus, PG = P. gymnonotus) and cave, with colors indicating ancestral population assignment. The lowest CV error was recovered for K = 3. [file 12862_2025_2465_MOESM1_ESM.png]

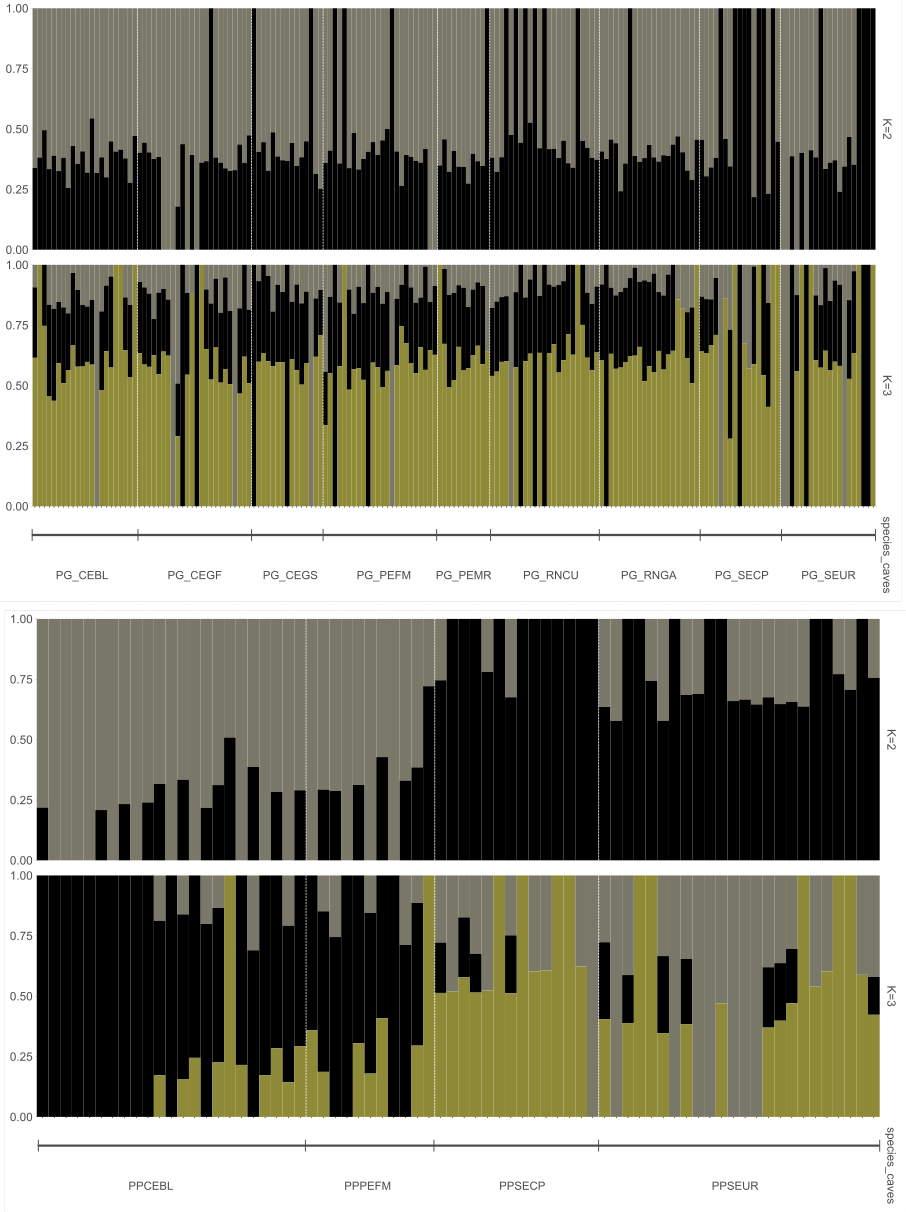

Supplement: Supplementary file 2 — Supplementary Material 2: Figure S2. ADMIXTURE results for P. gymnonotus and P. personatus. ADMIXTURE results for K=2 and K=3 for Pteronotus gymnonotus (top) and Pteronotus personatus (bottom) from bat caves in Northeast Brazil. Individuals are represented by horizontal bars, grouped according to the caves where they were sampled. [file 12862_2025_2465_MOESM2_ESM.tiff]
